# Citrate effect on the swelling behaviour and stability of casein microparticles

**DOI:** 10.1038/s41598-022-23096-x

**Published:** 2022-11-01

**Authors:** Md Asaduzzaman, Thomas Pütz, Ronald Gebhardt

**Affiliations:** Chair of Soft Matter Process Engineering (AVT.SMP), RWTH Aachen University, 52074 Aachen, Germany

**Keywords:** Biomaterials, Soft materials

## Abstract

Casein microparticles obtained from casein micelles by volume exclusion of added pectin and subsequent film drying remain stable in the acidic and neutral pH range, but swell strongly in the basic range. Calcium significantly impacts on the stability and water-binding behavior of phosphorylated caseins and the aggregates and gels formed from them. For a future effective and controlled use as a carrier for bioactive substances, e.g. via the gastrointestinal tract, we therefore investigated how the addition of the calcium chelating agent citrate affects the swelling and stability of the microparticles. Citrate concentrations of 2 mM and above cause a stronger swelling of the microparticles at pH 8, while above 4 mM the second characteristic swelling step starts earlier and thus can also be investigated within the observation time of 120 min. All swelling kinetics can be simulated using seven parameters of a dynamic model, which reproduces the individual swelling steps via volume inflows and outflows into a reservoir. While the rate coefficient for swelling step 1 increases linearly with citrate concentration, no such dependence could be found for swelling step 2. The more citrate is used, the faster the microparticles decompose in turbidity experiments after the addition of sodium dodecyl sulfate, which can be related to a weakening of the hydrophobic interactions.

## Introduction

Demand for biopolymer-based microparticles is continuously increasing. Microparticles are used, for example, as colloidal delivery systems in food, drug, and biomedical applications to encapsulate, protect and release bioactive ingredients. Protein-based biopolymers offer promising advantages over synthetically produced materials such as biocompatibility, biodegradability, low immunogenicity, antibacterial activity, lower risk of side effects, and low cost^1,2^. As a natural carrier of bioactives, milk proteins possess many structural and physicochemical properties to functionalize them for transport and delivery tasks^3^. Caseins are a particularly promising material for microparticulate delivery systems because they occur unfolded in the native state, have a high water- and calcium-binding capacity as well as a strong tendency to self-assemble into higher aggregated structures, and exhibit a high affinity for binding hydrophobic substances due to their amphiphilic nature^2,4^.

In bovine milk, four types of casein (α_S1_-, α_S2_-, β- and κ-casein) combine with calcium phosphates to form spherical association colloids with average sizes between 100 and 300 nm^5^. Caseins can be simplified as block copolymers to explain their adsorption behavior and assembly^6^. In this modeling approach, the caseins consist of alternating clusters of phosphoseryl-residues interacting with colloidal calcium phosphate and hydrophobic blocks through which caseins are interconnected. Colloidal stability is provided by κ-casein, which forms an extended polymer brush on the micellar surface^7^. Casein micelles are highly hydrated with about 3.3 g of water per g of protein, with a portion directly bound to the protein and others associated with the κ-casein polymer brush on the surface, but the major portion with 1.8 g/g is entrapped in voids or water-filled channels in the micellar structure^8^. A sponge-like internal structure with water-rich and water-poor regions was proposed as a model for casein micelles^5^ based on experimental studies^9,10^. Besides phosphate and chloride, citrate is a major anion in the aqueous phase of milk and acts similarly to polyphosphates and EDTA as a calcium chelator^11^. Indeed, added citrate demineralizes casein micelles by indirectly dissolving colloidal calcium phosphate^12,13^. After chelation has occurred, citrates form soluble complexes^14^, and no effects on intermicellar interactions, such as those that would lead to cross-linking or even gelation, have been observed^15^. As the interactions between phosphoseryl residues and calcium are reduced due to the action of citrate, the caseins become negatively charged, so their water-binding capacity is increased. However, significant changes in the voluminosity of the casein micelles only occur after the addition of 10 mEq L^−1^ chelator^15^. Studies with high hydrostatic pressure^16^ or by using denaturants such as sodium dodecyl sulfate (SDS)^17,18^ have demonstrated the importance of hydrophobic interactions for the internal structural stability of casein micelles.

Microparticles are produced in many different ways, including gelation, emulsification, spray drying or heat treatment^4,19–21^. Instead, we employed a gentle method of microparticles production based on depletion flocculation reaction in a casein-pectin system at pH 6.8^22,23^. When two casein micelles approach each other, their exclusion volumes for pectin overlap, so that polysaccharide is excluded from these areas. The volume exclusion of pectin and its accumulation outside the interaction region leads to a higher osmotic pressure in the surrounding area, which pushes the casein micelles together. The additional free volume arising from the overlap of the exclusion volumes results in an increase of the overall entropy of the system, which also entropically favors the attraction of casein micelles^24^. Under this process, the casein-pectin attractive interaction is not dominant but the volume exclusion by pectin is responsible for the formation of casein aggregates^22^. Stable microparticles were formed by film drying followed by enzymatic hydrolysis of the pectin film matrix. The produced CMPs were then suspended in buffer solution at pH 6.8. The resulting CMPs have a spherical shape, a prominent internal microstructure and are stable in BisTris buffer at 4 °C for 21 days^25^. Usually, other milk protein-rich hydrogel systems tend to degrade or swell at lower pH^26^. In the case of casein micelles, the acid-induced loss of colloidal stability leads to aggregation and gelation, so that they must be covalently linked to produce stable gel particles^27–29^. However, CMPs prepared by depletion flocculation reaction at neutral pH exhibit remarkable stability in an acidic environment. In contrast, CMPs swell under basic conditions in a two-step process, which takes a few seconds at pH 14, several minutes at pH 11, and hours at pH 8^30^. The first swelling step can be related to the expansion of the microstructure and the second step to the complete disintegration of the entire particle^25^. Casein films cross-linked with high methacrylated casein also showed stronger swelling in alkaline medium compared to those in acidic media^31^. In addition, the swelling kinetics also showed overshooting, which can also be observed with CMPs, but only if covalent cross-links with transglutaminase were subsequently inserted^32^. As the pH in the human stomach is highly acidic and near neutral in the duodenum^33^, the pH-dependent stability and swelling properties of CMPs can be favorable for the controlled release of the orally supplemented bioactive compounds. Because caseins are amphiphilic, they have the ability to bind both hydrophobic bioactive compounds such as vitamins^34,35^ and hydrophilic compounds such as polysaccharides. Moreover, the unfolded structure of caseins allows them to be more accessible to proteolytic enzymes in the gastrointestinal tract resulting in better release properties^3,4^. Biodegradable casein nanovehicles can be produced for oral delivery of poorly water-soluble drugs and be used, for example, in cancer therapy or to regulate intestinal flora^36,37^.

It is important to know the characteristic properties and stability of the produced microparticles before functionalization to protect and use the encapsulated material effectively. The microparticle produced under depletion flocculation has a highly porous inner structure that expands during swelling^38^. The concentration of CaCl_2_ in the running solution also has a significant effect on the swelling behavior of the microparticles. For instance, adding 0.1 mM CaCl_2_ at pH 11 slowed the two-phase linear growth of the particle area. In contrast, almost no swelling of CMPs was observed at a concentration of > 10 mM CaCl_2_ under the same pH conditions^25^. Free calcium and calcium phosphate nanoclusters facilitate cross-links between the negatively charged casein chains. The resulting neutralization of the charges allows stronger hydrophobic contacts between the caseins, which strengthens the internal particle structure^39^. The results of stability experiments with sodium dodecyl sulfate (SDS) indicate that hydrophobic interactions are essential for maintaining the overall structure, because CMPs decompose completely upon addition of the detergent^38^.

The application of casein microparticles for a sustainable delivery system of bioactive compounds is of great interest. Due to their characteristic pH-dependent swelling behavior, CMPs could be used in the future for microencapsulation through the gastric passage and controlled intestinal release of bioactive compounds. With the dosed use of the calcium chelating agent citrate, the charge state and the water-binding behavior of CMPs could be changed and thus their swelling behavior will be adjusted in a controlled manner. To the best of our knowledge, major studies have been reported describing the swelling behavior of casein micelles or casein microparticles produced by enzymatic cross-linking and other means as mentioned above. The present work focuses on improving the swelling behavior of microparticles, which have been prepared so far only by our group using depletion flocculation at pH 6.8 followed by film drying. Therefore, in this study, the effect of citrate on the stability and swelling behavior of CMPs at two relevant pH values was investigated to tailor the functionality for future applications.

## Material and methods

### Materials

Casein micelle concentrate (CMC) powder MC 80 was kindly provided by Milei GmbH Germany and citrus pectin (classic CU 201) was from Herbstreith & Fox (Herbstreith & Fox GmbH & Co. KG, Neuenbürg, Germany). Pectinase from *Aspergillus niger*, tri-sodium citrate, hydrochloric acid (1 M), sodium hydroxide (1 M), and sodium azide was from Merck (Merk, Darmstadt, Germany). Especially pure sodium dodecyl sulfate (SDS), ultra-pure BisTris, calcium chloride, and all salts (purity > 99% or analytical grade) for SMUF preparation were obtained from VWR, Radnor, USA. Milli-Q water was obtained from our lab.

### Preparation of working solutions

BisTris buffer solution (50 mM BisTris, 10 mM CaCl_2_) was prepared by adding 10.462 g BisTris in a volumetric flask containing about 980 mL milli-Q water under continuous stirring. After complete dissolution, 1.11 g CaCl_2_ was added to the solution and allowed to dissolve completely. The pH of the solution was adjusted to 6.8 with 1 M HCl and NaOH and finally the volume was increased to 1 L.

Simulated milk ultra-filtrate (SMUF) solution was prepared according to a protocol described by Dumpler^40^. All salts were dissolved step by step, allowing the complete dissolution of the previous salt before the next salt was added. Finally, pH was adjusted at 6.8 with 1 M HCl and NaOH.

Pectin solution (2%) was prepared by dissolving 1 g pectin in 49 g BisTris buffer solution with vigorous stirring at 80 °C for 3 h and then cooled down to room temperature. Finally, pH was adjusted to 6.8 with 1 M HCl and NaOH.

Casein dispersion (7.36%) was prepared by dissolving 2 g of CMC powder (protein > 80% of which > 92% casein) in 18 g of SMUF. The CMC powder was allowed to dissolve under continuous stirring at room temperature for 1 h followed by 4 h at 4 °C and finally 1 h at 37 °C. To avoid microbial contamination, 0.5 g/L sodium azide was added to the dispersion. The addition of sodium azide had no effect on the stability of the CMPs, as shown by the results in Supplementary Fig. S1.

Pectinase solution (activity ≈ 36 units/mL) was prepared by adding 0.47 mL pectinase (activity ≈ 800 units per mL) from *Aspergillus niger* to 10 g BisTris buffer and mixed properly. The solution was prepared immediately before adding to the film.

### Preparation of Casein microparticles (CMPs)

**Figure 1:**
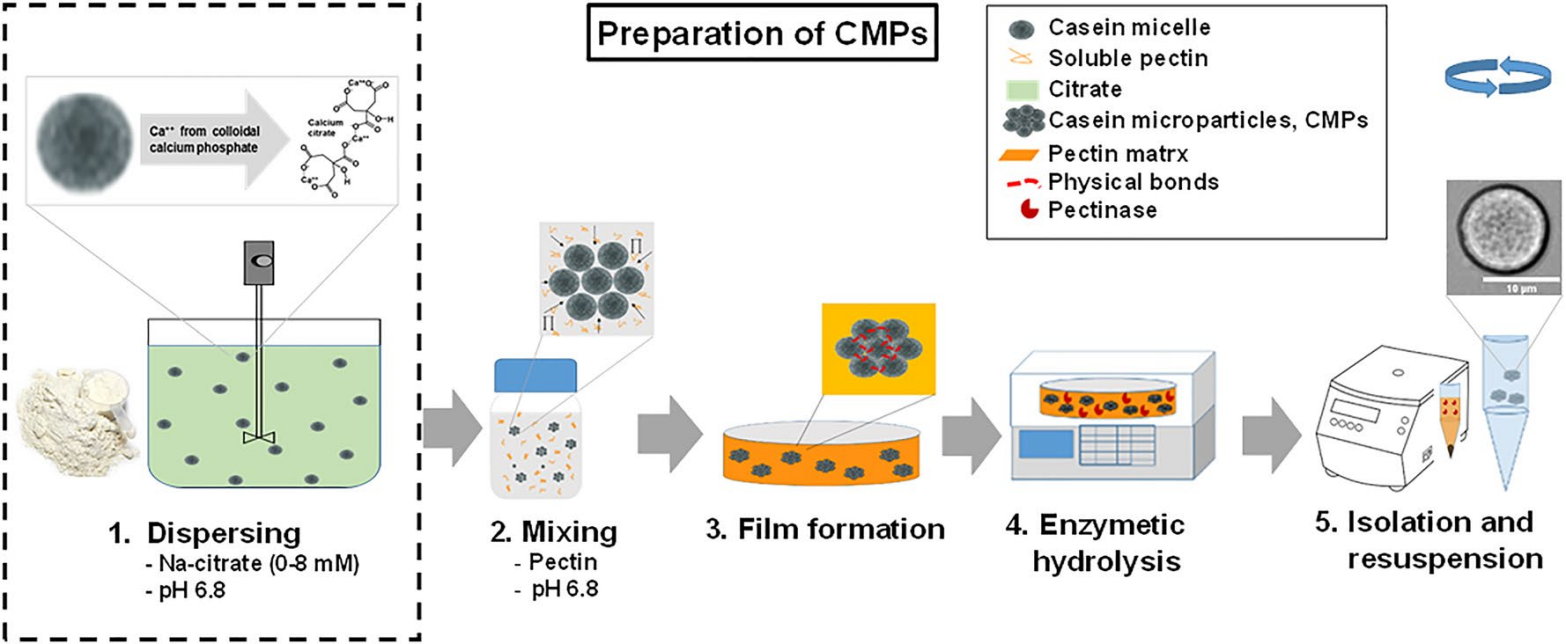
Process steps of producing casein microparticles at pH 6.8.

The principle of the CMPs production process was based on the depletion flocculation interaction between casein micelles and pectin under neutral pH-conditions^22^. The individual preparation steps are summarized in Fig. 1 and the corresponding photographs and microscopic images in Supplementary Fig. S2. The preparation of the CMPs was according to a protocol described by Schulte^30^. Briefly, the casein solution, pectin solution, and BisTris buffer solution mentioned above were mixed properly in a proportion of 4.1:1.5:4.4 (w/w) to obtain 3.0% casein and 0.3% pectin in the final solution^30^. Then 3.9 g of this mixed solution was transferred to a glass petri dish (Ø 70 mm) and dried at room temperature for 16 h to produce a film. To hydrolyze this film 10 g pectinase solution was added to the petri dish. The enzymatic hydrolysis was performed in a ThermoMixer (Eppendorf, Eppendorf AG, Hamburg, Germany) at 47 °C for 2 h with 160 rpm. After hydrolysis, the supernatant solution was collected in a falcon tube and centrifuged at 22 °C and 1500 RCF for 10 min. The clear solution was poured out from the top and the resulting stabilized pellet of CMPs was then suspended again in BisTris buffer and stored at 4 °C for further use. For the preparation of citrate-treated casein microparticles (0 to 8 mM), the required amount of tri-sodium citrate solution (200 mM in water) was added to the casein dispersing solution, while the other steps of the preparation remained the same as described above.

### Swelling experiments

The swelling behavior of CMPs was studied according to the protocol developed by Schulte^30^. Briefly, the swelling chamber was filled with CMPs dispersion (in BisTris buffer, pH 6.8) and placed under Leica DMIL LED inverted microscope (Leica Microsystems, GmbH, Wetzlar, Germany) connected with a Basler camera (Basler AG, Ahrensburg, Germany). The dispersion was allowed to stand for approx. 10 min to sediment the CMPs into the sieve holes. A PHD ULTRA™ syringe pump (Harvard Apparatus, MA, USA) was connected to the swelling chamber by polyethylene tubes (Ø 0.55 mm). The pump flow rate was set at 0.05 mL per min for the exchange medium (ultrapure water, pH 3 or 8). The swelling process of the CMPs at different pH was started by replacing the buffer solution with an exchange medium. With the activation of the syringe pump, an image of a single microparticle trapped in the sieve holes was set to record (at the rate of 2 frames per second for 2 h) using the Basler video recording software. Image frames were extracted using PyCharm (version 2021.1.3, JetBrains, Czech) and the area of the CMPs was calculated by a freehand selection of particle outer lines using ImageJ software (NIH, USA). All samples were measured in duplicate.

### Dynamic swelling model and data analysis

For a more detailed analysis of the swelling behavior, the cross-sections of the CMPs in the micrographs were first evaluated and plotted as a function of swelling time. The influence of citrate on the typical two-stage swelling behavior of the CMPs can be simulated with a dynamic model whose basic structure has already been described^25^. Volumes are first calculated from the determined cross-sectional areas for the simulation, assuming the spherical approximation that is satisfied for the swelling process^38^. Within the model, the volume changes by two inflows and two outflows respectively, which are controlled by valves. All incoming and outgoing volume flows are proportional to the current volume of the CMPs at each time point. The rate coefficients for the inflows and outflows assume a time-invariant value after a characteristic time through a step function (or through a transition function as exemplified in Fig. 3). The model is able to describe all measured swelling kinetics to a good approximation by a maximum of 9 parameters (4 characteristic times and 4 rate coefficients as well as an initial value for volume or particle area). The numerical integration of the underlying differential equations was performed with the program Stella, isee systems.

### Stability experiments

For stability analysis, the turbidity of CMPs solution was monitored using a Lambda 365 UV/VIS spectrometer (PerkinElmer, USA) according to the method described by Schulte^38^ with slight modification. Briefly, 1.5 mL CMPs dispersion was added to a semi-micro cuvette (Eppendorf AG, Germany) and BisTris buffer was used as reference. The turbidity was measured at a wavelength of 600 nm, slit width of 1 nm and absorbance was recorded for 900 s. Immediately after measurement, 40 µl SDS solution (520 mM in water) was added to CMP dispersion resulting in a 13.5 mM final concentration of SDS. The cuvette was then gently moved up and down 3 times to properly mix the SDS, placed back in the spectrometer, and measured again. All samples were measured in duplicate.

## Results and discussion

**Figure 2:**
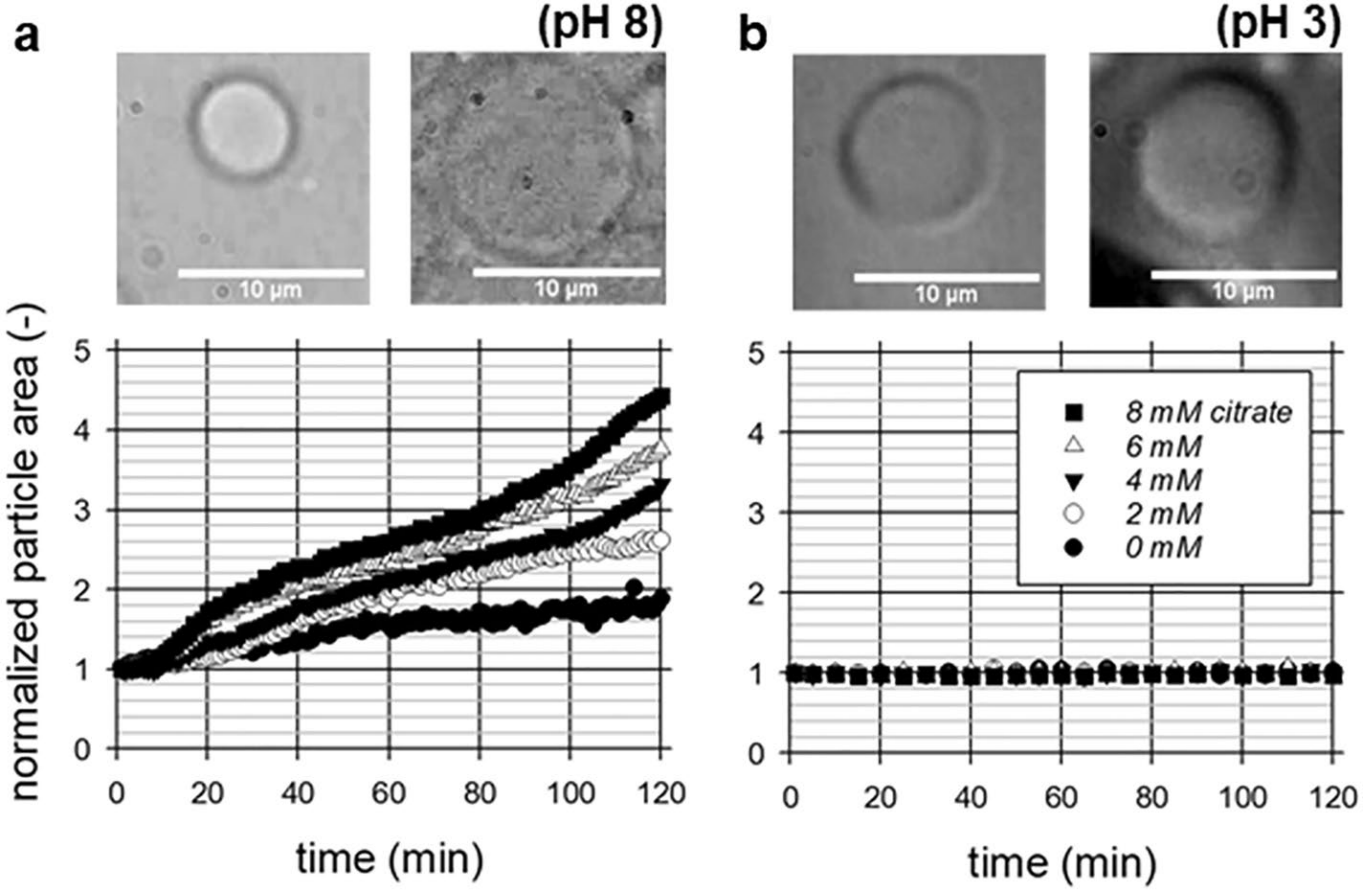
Microscopic images of CMPs with 8 mM citrate at the beginning and 120 min after pH change to pH 8 (**a**) and pH 3 (**b**) occurred, and normalized swelling curves at different citrate concentrations each obtained by averaging two individual kinetics of the particle areas.

We prepared CMPs with different citrate concentrations at pH 6.8 and performed swelling and stability experiments with the resulting particles at pH 8 and pH 3. SDS stability studies have shown that optimal treatment is achieved when citrate is added in the first step of the preparation process shown in Fig. 1 (see stability data in Supplementary Fig. S3). The microscopic images in Fig. 2 show the circular cross-sections of the particles in the used swelling cell at the beginning and 120 min after the pH change to pH 8 and to pH 3, respectively. While there is a significant increase in cross-sectional area at pH 8, no change can apparently be seen at pH 3, which we also observed in a previous study^30^. Modified casein micelles also show an increase in size in alkaline medium due to increasing electrostatic repulsion, but in contrast shrink after acidification due to dissolution of colloidal calcium phosphate and reorganization of the internal structure^41,42^. Interestingly, we have also observed acid-induced shrinkage in regenerated fibers from rennet-treated casein micelles^43,44^. For CMPs, however, it has been reported that caseins are already in a very compact gel state due to the manufacturing process, so that possible structural changes after acidification cannot be resolved^30^. Citrate is known to indirectly demineralize casein micelles by chelating ionic calcium in the aqueous phase^11,45^. As a result of the residual negative charge on the casein and the osmotic contribution of increased content of counter ions, the casein micelles become more hydrated and swollen^13,15,46^. This results in increased swelling at pH 8 but not at pH 3. Under the acidic conditions, it can be assumed that the structure of caseins in CMPs hardly changes. For comparison, individual casein micelles form an acid-induced gel below a pH of 5.2 because the stabilizing κ-casein surface layer collapses^47^. In addition, below the isoelectric point of casein (at pH 4.8), many amino acid residues of casein are present in a protonated state, resulting in strong hydrogen bonds that compress the structure and prevent swelling^48^. However, at pH 8 the citrate-induced increase in electrostatic repulsion and the higher solvent quality of the environment lead to weaker cohesive interactions between the hydrophobic regions of the neighboring caseins^49^. As a result, expansion occurs and the newly formed cavity volume is filled with solvent molecules of the surrounding environment^9,46^. For detailed analysis, the cross-sectional areas of the CMPs from the image sequences of two individual swells were evaluated and averaged, and plotted in the swell curves below.

The particle areas were related to those at the beginning of the swelling process where no exchange buffer was present in the swelling cell. A clear citrate effect on the degree of swelling and the course of the overall process can be seen based on the swelling kinetics. While the CMPs without citrate do not even double in particle size after 120 min, a maximum degree of swelling was achieved within this observation window for preparations with 8 mM citrate. For the CMPs prepared with less citrate, the degree of swelling at the end of the kinetics increases almost linearly with the citrate concentration. For CMPs without citrate influence, we have previously reported a two-step swelling process that finally resulted in the complete dissolution of the particles^30^. We found that the particle area increased almost linearly with swelling time during both swelling steps, but first at a lower and then at a higher rate. While both steps of the overall process could be resolved well in the range of pH 11 to pH 14, this has only been possible to a limited extent at pH 8 due to the time-consuming swelling process under these relevant milieu conditions. In detail, only limited swelling data could be collected for swelling step 2 until now at swelling times > 100 min. However, with citrate treatment, the second swelling step of the CMPs can now be resolved. For swelling curves at 4, 6 and 8 mM citrate, it starts after about 100 min with nearly the same rate in each case. The rate of the first swelling step, however, increases more and more with citrate concentration. In contrast to the experiments without citrate addition, swelling step 2 does not occur immediately following swelling step 1. Instead, we observe an intermediate time period where the particle area increases less. This occurs, for example, in the swelling curve for 8 mM citrate at times between 30 and 100 min. We observed a similar phase of reduced swelling in the kinetics at pH 11 when 0.1 mM calcium or higher was added to the exchange buffer^25^. In this case, the addition of calcium results in further physical linkages that slow down and limit the swelling process.

**Figure 3:**
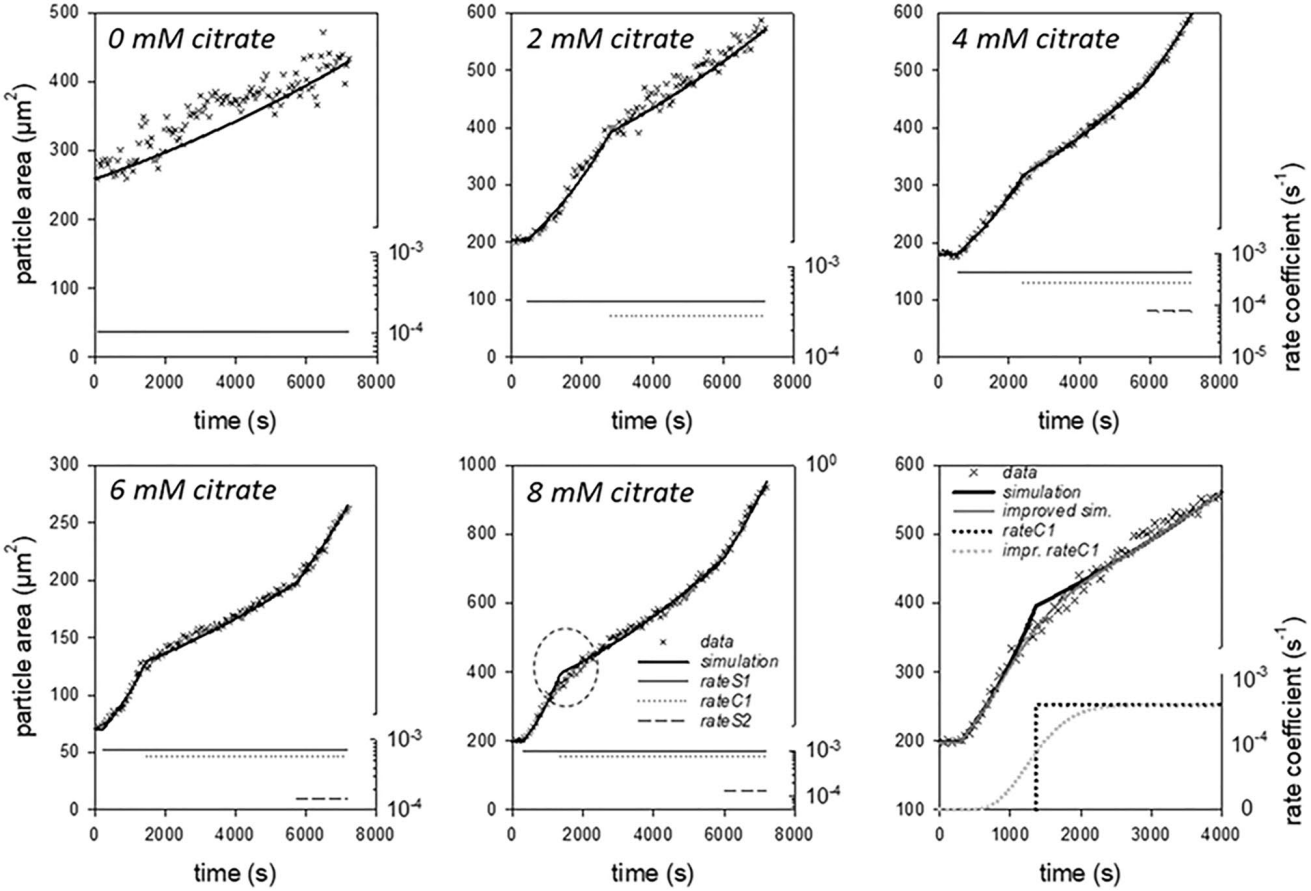
Individual swelling kinetics at pH 8 for CMPs, which were prepared without and with 2, 4, 6 and 8 mM citrate. The data correspond to the absolute particle areas and the lines are simulations with the dynamic swelling model. The values of the rate coefficients used from certain characteristic times via a step function to set the volume flows are also shown.

For a more detailed analysis, we simulated the swelling kinetics with a dynamic model, which describes the different phases of particle swelling via different volume flows with respect to a reservoir. The phases of reduced expansion following the swelling steps which were observed after the addition of calcium, or in this case under the influence of citrate, can be simulated by additional volume flows out of the reservoir. The structure of the dynamic model used is described in detail under Materials and Methods. Figure 3 shows one of the swelling kinetics of CMPs for each of the citrate concentrations and for the reference without citrate addition along with the model simulations. In total, up to seven parameters were adjusted for the simulation of the kinetics.

The swelling kinetics of the CMPs without citrate influence could be simulated using only one swelling step with a constant rate coefficient and a corresponding characteristic starting time. Under these conditions, there is no evidence of flattening of the swelling of the first step nor of the existence of a second swelling step, which may be due to both the comparatively large scatter of the data and the limited observation period of 7200 s. In contrast, CMPs prepared with 2 mM citrate swell faster but also exhibit a significantly longer lag phase before the swelling step begins. However, there is an abrupt change in the increase in particle area during swelling. After about 3000 s, the particles swell significantly slower with a reduced but constant rate. The corresponding feature in the swelling curve can be simulated by a volume outflow with a characteristic rate coefficient over another step function with a time offset (see dotted line for the corresponding rate coefficient).

In the swelling kinetics of the CMPs prepared with citrate concentrations of 4–8 mM, a second swelling step can be seen in addition to the first swelling step and subsequent region of reduced expansion. To simulate these swelling data, a volume inflow and outflow for swelling step 1 and a volume inflow for swelling step 2 were used with the corresponding rate coefficients for the time ranges plotted in the lower part of the graphs. Due to the jump functions used for the rate coefficients, discontinuities such as kinks occur in the simulations. Such a kink appears, for example, for the swelling of CMPs prepared with 8 mM citrate during the first swelling step at the transition to the decelerated phase (see dotted ellipse). The corresponding area of the swelling curve is magnified in the plot at the bottom right of Fig. 3. The simulation of the measured data can be significantly improved by adding another model parameter for the use of a transition function instead of a step function for the rate coefficient. Due to the internal structuring of the CMPs, it can be assumed that the physicochemical properties of a large number of structural elements determine the swelling process. In particular for the first swelling step, we assume that this is based on the expansion of a few µm-sized building blocks within the CMPs. Since their composition and size are not uniform but distributed, it can be assumed that a changed swelling behavior follows a finite transition rather than a sharp step function.

**Figure 4:**
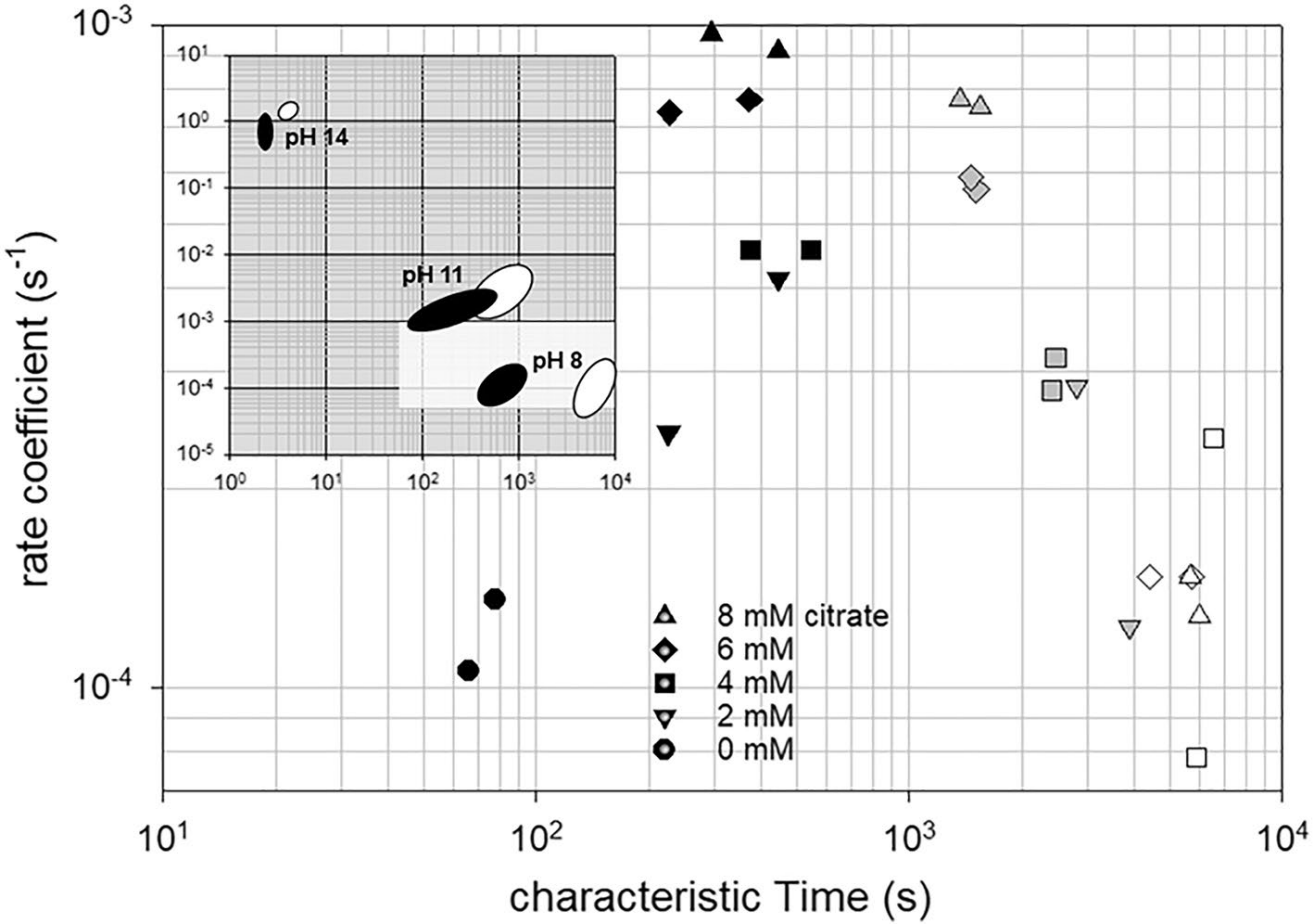
Swelling coefficients as a function of their characteristic start times for the first step (closed symbols) for the subsequent slowed phase (grey symbols) and for the second step (open symbols) of swelling of CMPs at pH 8.0 prepared without or with different citrate concentrations. The insert shows how the location of the parameter pairs for the first and second swelling steps of CMPs without citrates (black and white areas) changes as a function of pH (reconstructed from Schulte^30^).

**Figure 5:**
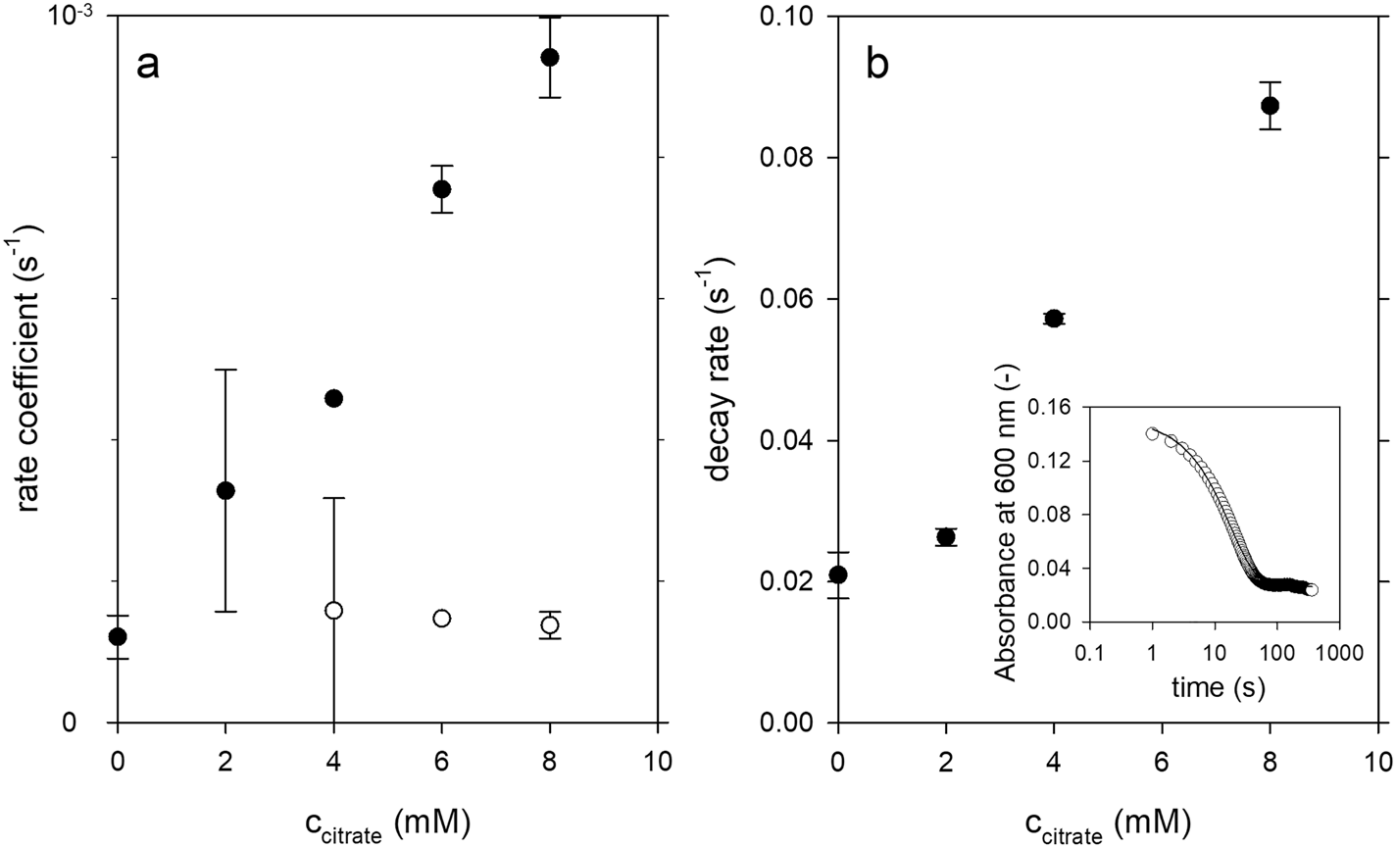
Rates determined from the swelling curves (**a**) and SDS-turbidity experiments (**b**) of CMPs at pH 8 as a function of the citrate concentration used to prepare the particles. Closed symbols in (**a**) correspond to the rate coefficient of swelling step 1 and open symbols correspond to the rate coefficient of swelling step 2.

The rate coefficients used for the simulation of the individual swelling steps are plotted over their corresponding characteristic times for all measured kinetics in Fig. 4. For comparison, the ranges of values obtained for the pH-dependent swelling of CMPs prepared without citrate are shown in an inset. For these reference samples, the swelling curves consisted of only two directly consecutive swelling steps. The double logarithmic plot shows that particularly high swelling rates and short times to the onset of each swelling step occur at pH 14, while the reverse is true at pH 8. All kinetic data at pH 8 collected in this study lie in the white-marked region and are thus clustered around the previously collected data without citrate at pH 8^30^. The aim of using citrates was both to increase the swelling rates and to shorten the characteristic times until the onset of the swelling processes. Transferred to the insert in Fig. 4, this means a diagonal shift of the value ranges from the lower right at pH 8 to the upper left in the direction of pH 11. As can be seen from Fig. 4, under citrate influence, the rate coefficients for the first swelling step actually shift to higher values. In fact, we observe a linear increase of this rate coefficient as a function of citrate concentration as Fig. 5a shows. In contrast, there appears to be no citrate dependence for the rate coefficient of the second swelling step, as the values fluctuate around a constant value. While the first swelling step can be assigned to the expansion of casein micelles or µm-sized building blocks, the second swelling step results from the disruption of the contacts between the individual building blocks, which finally leads to the disintegration of the CMPs^25^. The pronounced citrate dependence of the first swelling step can therefore be attributed to the degradation of the colloidal calcium phosphate present within the casein micelles and building blocks. In contrast, the presence of colloidal calcium phosphate between the building blocks is rather unlikely, which is confirmed by the citrate independence of the second swelling step. The characteristic times, on the other hand, shift to higher values within the newly acquired data for the first swelling step, while no statements can be made for the second swelling step.

Thus, contrary to the assumptions, the time until the start of the first swelling step is not shortened by the citrate influence, but delayed by a few minutes. Furthermore, similar to the pH effect, citrate influences the rate coefficients for both swelling steps relative to each other. This can be seen by comparison with the reference samples analyzed at different pH values but without citrate influence in the insert in Fig. 4. While at pH 14 the rates of the second swelling step (open areas) are clearly larger than those of the first swelling step (black areas), this difference is less pronounced at pH 11. At pH 8, the areas of values for the rate coefficients of both steps are at the same level. After the addition of citrate, this trend is now continued, although the proton concentration is kept constant at pH 8. For all CMPs with citrate influence, the rates of the second swelling step are lower compared to the first step. The reason for this is the positive citrate dependence of the first swelling step, which does not exist for swelling step 2 as shown in Fig. 5a. In addition to stronger swelling, we also observe increasing instability of the CMPs with increasing citrate concentration. This is indicated by turbidity experiments that resulted in monoexponential decay for all particle preparations after SDS addition, as exemplified as an inset in Fig. 5b. The decay rates obtained after fitting with an exponential function indicate more unstable CMPs with citrate. The destabilization can be attributed to a weakening of the hydrophobic interactions between the caseins^17,18^. Since citrate primarily removes calcium from the caseins, which leads to a negative charge on the caseins, the weakening is probably a secondary effect caused by increasing electrostatic repulsion^50^.

Several works have reported the stability and swelling behavior of casein micelles^8,13,15,45,46,48,49^ or casein microparticles produced by enzymatic cross-linking^12,13^ and other means^19,26,30^. However, the present study has demonstrated the effect of citrate to enhance the swelling behavior of casein microparticles produced by volume exclusion of added pectin and subsequent film drying. The produced CMPs were stable in the acidic medium but swelled faster in the basic range with the dose-dependent addition of citrate. Experiments with pH-sensitive cross-linked casein films have shown that faster swelling in the alkaline range also leads to higher delivery rates of the model drugs serum albumin and ofloxacin^31^. Because of their characteristic pH-dependent swelling behavior, CMPs could be used in the future as microcapsules for the protection of bioactive compounds in acidic medium and for controlled release in alkaline medium. With the help of dosed citrate during production, the release behavior could be adjusted in a controlled manner.

## Conclusions

CMPs show a strongly reduced swelling behavior at the physiologically important milieu condition of pH 8. On average, the swelling process must be observed under the microscope for two hours until the particle areas of the CMPs double. The aim of this study was to improve the swelling behavior by destabilizing the starting material, i.e. the casein micelles, by using citrate during the manufacturing process of the CMPs. Our studies show a clear citrate effect on the swelling behavior and stability of the CMPs. When 8 mM citrate is used during preparation, we observe that the particle areas of the resulting CMPs swell to more than four times their initial size at pH 8. Moreover, our results show that the more citrate is used, the faster the first step of the two-step swelling process occurs. In addition to the occurrence of a phase with reduced swelling velocity within the first swelling step, we further observe that under the influence of citrate the entire swelling process accelerates to such an extent that the second swelling step can also be investigated. The influence of citrate on casein micelles is already well studied, which leads to the degradation of colloidal calcium phosphate as a result of the Ca-chelating effect of citrate. The results shown here regarding the influence of citrate on the swelling steps confirm a swelling model for the CMPs recently proposed by us^25^. During the first swelling step, the microstructure within the CMPs initially expands. The corresponding swelling rate increases with the concentration of citrate, as the colloidal calcium phosphate of the casein micelles is destabilized. However, in the second swelling step, the contacts between the building-blocks, which are only formed during the CMPs production process, are loosened. At these contact sites, the occurrence of colloidal calcium phosphate is rather unlikely, which is confirmed by the citrate independence of the corresponding swelling rate. Within CMPs, the citrate effect could be explored in the future by confocal fluorescence lifetime imaging microscopy via the detection of free calcium concentration, which we have also recently determined in regenerated fibers from rennet-treated casein micelles^43^. Future work should investigate whether the swelling behavior of TGase-treated CMPs, which is reduced by cross-links, can be enhanced by the use of citrate during the manufacturing process. The improved swelling behavior at pH 8 and the lower stability of CMPs prepared with citrate might also favor the release of the encapsulated substances under basic conditions in the intestine. For this purpose, however, the influence of proteolytic enzymes on the stability and swelling behavior of citrate-treated CMPs in real gastric and intestinal fluids remains to be investigated.

## Supplementary Information

Supplementary Information.

## Data Availability

The datasets generated during the current study are available from the corresponding author on reasonable request.
