# Supplementary material for: Citrate effect on the swelling behaviour and stability of casein microparticles
Source: Sci Rep. 2022 Nov 1;12:18401. doi: 10.1038/s41598-022-23096-x (PMC9626602; doi:10.1038/s41598-022-23096-x)
Supplement: Supplementary file 1 — Supplementary Information. [file 41598_2022_23096_MOESM1_ESM.docx]

**Supplementary Information for**

**Citrate Effect on the Swelling Behaviour and Stability of Casein Microparticles**

Md Asaduzzaman^1^, Thomas Pütz^1^, Ronald Gebhardt*^1^

^1^ RWTH Aachen University, Soft Matter Process Engineering (AVT.SMP), 52074 Aachen, Germany

*Corresponding author: ronald.gebhardt@avt.rwth-aachen.de

M.A.: https://orcid.org/0000-0002-6633-1289

R.G.: https://orcid.org/0000-0002-9702-1996

1. Effect of sodium azide on the stability of CMPs

We investigated the effect of sodium azide on the SDS stability of CMPs using turbidity measurements. The decay rates obtained are shown in Fig.S2.

**Fig. S1** Effect of sodium azide (0.05 g L^-1^) on the stability of CMPs

We found that CMPs prepared with or without sodium azide exhibited similar stability toward SDS, so that an effect on the structure and function of CMPs by azide can be excluded.

1. Preparation process of CMPs


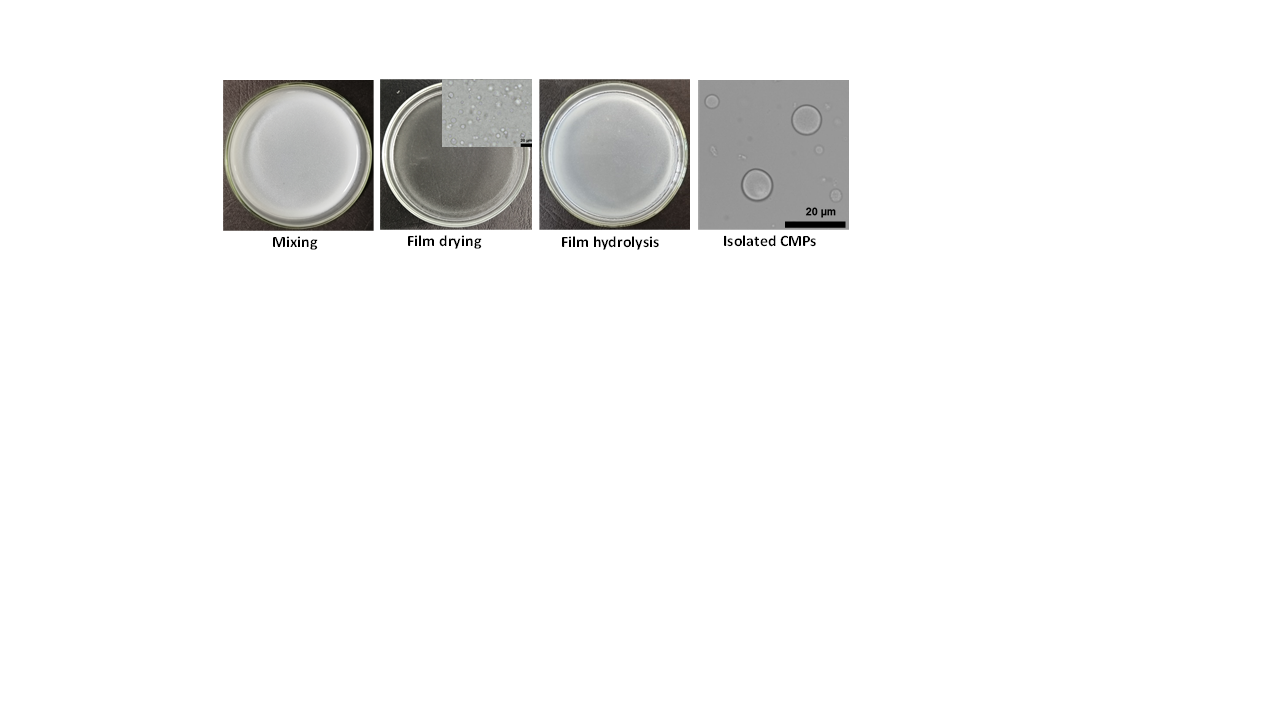


**Fig. S2:** Related fotos and micrographes

1. Addition of citrate at various stages of CMPs preparation

We added citrate at different stages of the CMP preparation process to find the optimal conditions for the destabilization effect. We then performed stability studies to investigate the effects of citrate addition on CMPs prepared by this method. The obtained decay rates are shown in Fig.S1. CMPs prepared by adding citrate during the preparation of the casein-pectin mixture (see step 2 in Fig. 1) showed similar stability as the control sample without citrate (sample **C** and **A**). In contrast, the addition of citrate to the casein micelles (step 1 in Fig. 1) resulted in increased destabilization (sample **B**). Furthermore, the addition of citrate after CMPs preparation (step 5 in Fig. 1) resulted in the strongest destabilization and decomposition of CMPs (sample **D**). Therefore, we decided to add citrate to the casein solution in step 1 to investigate its effect on the swelling behaviour of the CMPs.


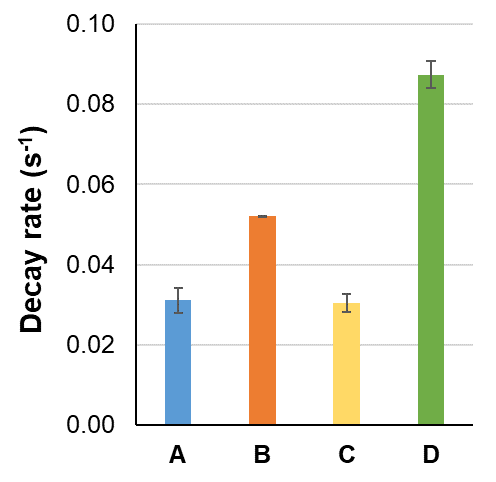


**Fig. S3** Effect of citrate addition (6 mM) at different stages of the CMPs manufacturing process shown in Fig.1. The decay rates for A: without citrate, B: citrate addition into the casein solution, C: citrate addition during the preparation of the casein-pectin mixture, D: citrate addition after CMP preparation are shown as bars in different colors
